# Supplementary material for: Modeling the efficiency of filovirus entry into cells in vitro: Effects of SNP mutations in the receptor molecule
Source: PLoS Comput Biol. 2020 Sep 28;16(9):e1007612. doi: 10.1371/journal.pcbi.1007612 (PMC7544041; doi:10.1371/journal.pcbi.1007612)
Supplement: S1 Text — (DOCX) [file pcbi.1007612.s001.docx]

**Supplementary Information**

Modeling the efficiency of filovirus entry into cells *in vitro*:

Effects of SNP mutations in the receptor molecule

Kwang Su Kim^1,†^, Tatsunari Kondoh^2,†, ♦^, Yusuke Asai^3^, Ayato Takada^2^, Shingo Iwami^1,4,5,6‡,^*

^1^Department of Biology, Kyushu University, Fukuoka 8128581, Japan. ^2^Research Center for Zoonosis Control, Hokkaido University, Hokkaido 0010020, Japan. ^3^Disease Control and Prevention Center, National Center for Global Health and Medicine, Tokyo 1628655, Japan. ^4^MIRAI, Japan Science and Technology Agency, Saitama 3320012, Japan. ^5^CREST, Japan Science and Technology Agency, Saitama 3320012, Japan. ^6^Science Groove Inc., Fukuoka 810-0041, Japan.

♦Current address

Tatsunari Kondoh: Tsukuba Branch, WDB Co., Ltd., 1-6-1 Takezono, Tsukuba, Ibaraki, 305-0032, Japan

† These authors contributed equally to this study.

* Correspondence and requests for materials should be addressed to S.I. ([siwami@kyushu-u.org](mailto:siwami@kyushu-u.org)).

**S1 Text: Simulation of merging viral plaques**

We sometimes observed that several plaques expanded to form a contiguous plaque in our plaque assay. Here, we introduced a method to represent merging plaques using our mathematical model, Eqs. (1-5). Let us assume that each plaque amplification occurs at the same speed and the origin of plaque is fixed as $(0,0)$ and $(2a,0)$, that is, representing the merging two plaques with identical radii. When the circles radius defined as $r$ from $(0,0)$ and $(2a,0)$ becomes large, two plaques intersect one another and overlapping occurs if $r>a$ (see **S1A Fig.**). The intersection of $A$ is calculated as $(a,\sqrt{r^{2}-a^{2}})$, which can be converted into a polar coordinate $\theta\left( r \right)$. The angle excluding the part at which the circles overlap is defined as $p\left( r \right)=1-2\theta(r)/2\pi$ if $r>a$: otherwise $p\left( r \right)=1$. For our approximate calculations, therefore, we assumed that the infectious phase cells excluded the overlap part and were located at the radius $r-\Delta r$, $I\left( t,r-\Delta r \right)p(r-\Delta r)$, can infect uninfected cells and excluded the overlap part at the radius of $r$, $T\left( t,r \right)p(r)$. Thus, our mathematical model considering two plaques merging is modified as follows:

$$\frac{dT\left( t,r \right)}{dt}=-\alpha\omega p(r)T\left( t,r \right)\sum_{j=1}^{n_{I}} {p(r-\Delta r)I}_{j}\left( t,r-\Delta r \right), \left( S1 \right)$$

$$\frac{dE\left( t,r \right)}{dt}=\alpha\omega p(r)T\left( t,r \right)\sum_{j=1}^{n_{I}} p(r-\Delta r)I_{j}\left( t,r-\Delta r \right)-kE\left( t,r \right), \left( S2 \right)$$

$$\frac{dI_{1}\left( t,r \right)}{dt}=kE\left( t,r \right)-\frac{n_{I}}{\tau_{I}}I_{1}\left( t,r \right), \left( S3 \right)$$

$$\frac{dI_{j=2,\ldots,n_{I}}\left( t,r \right)}{dt}=\frac{n_{I}}{\tau_{I}}I_{j-1}\left( t,r \right)-\frac{n_{I}}{\tau_{I}}I_{j}\left( t,r \right), \left( S4 \right)$$

$$\frac{dD\left( t,r \right)}{dt}=\frac{n_{I}}{\tau_{I}}I_{n_{I}}\left( t,r \right). (S5)$$

The initial conditions were: $I_{1}\left( 0,r \right)=2\pi\times r/0.008$ for $r\leq r_{0}$ and $0$ for $r>r_{0}$, $T\left( 0,r \right)=0$ for $r\leq r_{0}$ and $2\pi\times r/0.008$ for $r>r_{0}$, and $E\left( 0,r \right)=D\left( 0,r \right)=0$ for $r>0$. This approach is easily extended to the merging of two plaques with different radii and the merging of three (and more) plaques as well. For example, in the case of merging plaques with circle radii of $r_{1}$ and $r_{2}$ from their origin of $(0,0)$ and $(2a,0)$ described in **S1B Fig.**, the intersection of $A$ is calculated as $(\alpha,\sqrt{r^{2}-\alpha^{2}})$ where $\alpha={(r}_{1}^{2}-r_{2}^{2}+4a^{2})/4a$. Using polar coordinates $\theta\left( r_{1} \right)$ and $\theta\left( r_{2} \right)$, we can similarly simulate merging viral plaques with the angle excluding the part at which the circles overlap of $p\left( r_{1} \right)=1-2\theta(r_{1})/2\pi$ and $p\left( r_{2} \right)=1-2\theta(r_{2})/2\pi$ if $r_{1}+r_{2}>2a$; otherwise $p\left( r_{1} \right)=p\left( r_{2} \right)=1$.

**Figure legends**

**S1 Fig.** **Ratio of plaque overlap during plaque merging:** Overlapping angles of two plaques with the same circle radius is described in **(A)** and with different radii is described in **(B)**.

**S1 Fig.** **Ratio of plaque overlap during plaque merging**

**S1 Movie.** **Spatial-temporal dynamics of two plaques merging**

**S2 Movie.** **Spatial-temporal dynamics of three plaques merging**
