# Supplementary material for: Modeling the efficiency of filovirus entry into cells in vitro: Effects of SNP mutations in the receptor molecule
Source: PLoS Comput Biol. 2020 Sep 28;16(9):e1007612. doi: 10.1371/journal.pcbi.1007612 (PMC7544041; doi:10.1371/journal.pcbi.1007612)

**S1 Figure.** **Ratio of plaque overlap during plaque merging:** Overlapping angles of two plaques with the same circle radius is described in **(A)** and with different radii is described in **(B)**.


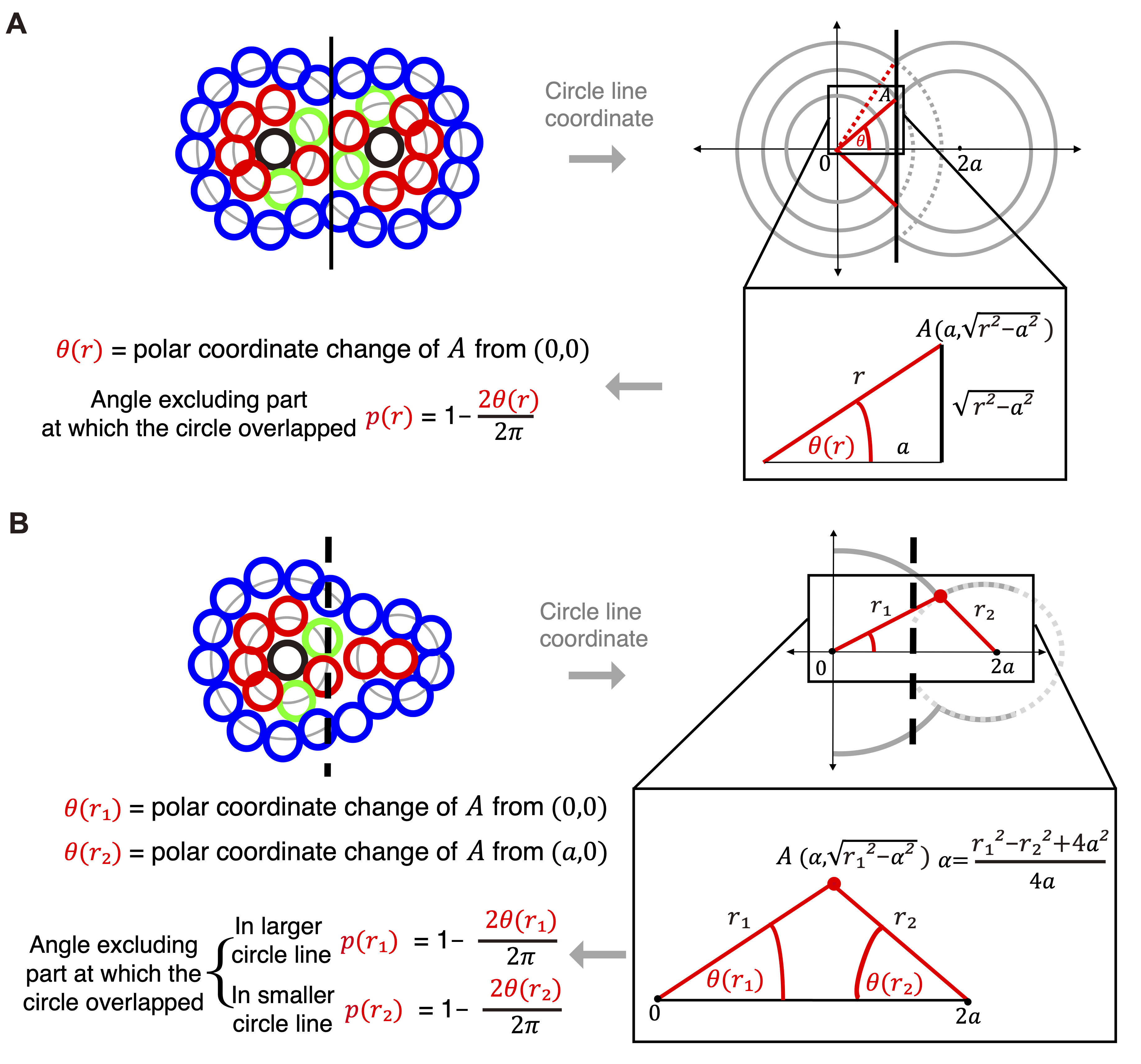

Supplement: S1 Fig — (DOCX) [file pcbi.1007612.s002.docx]
